# Serotonin Increases Cilia-Driven Particle Transport via an Acetylcholine-Independent Pathway in the Mouse Trachea

**DOI:** 10.1371/journal.pone.0004938

**Published:** 2009-03-17

**Authors:** Peter König, Benjamin Krain, Gabriela Krasteva, Wolfgang Kummer

**Affiliations:** 1 Institut für Anatomie, Zentrum für medizinische Struktur- und Zellbiologie, Universität zu Lübeck, Lübeck, Germany; 2 Institut für Anatomie und Zellbiologie, University of Giessen Lung Center, Justus-Liebig-Universität Giessen, Giessen, Germany; Emory University, United States of America

## Abstract

**Background:**

Mucociliary clearance in the airways is driven by the coordinated beating of ciliated cells. Classical neuromediators such as noradrenalin and acetylcholine increase ciliary beat frequency and thus cilia-driven transport. Despite the fact that the neuromediator serotonin is ciliostimulatory in invertebrates and has been implied in releasing acetylcholine from the airway epithelium, its role in regulating cilia function in vertebrate airways is not established.

**Methodology/Principal Findings:**

We examined the effects of serotonin on ciliary beat frequency and cilia-driven particle transport in the acutely excised submerged mouse trachea and determined the sources of serotonin in this tissue by immunohistochemistry. Serotonin (100 µM) increased cilary beat frequency (8.9±1.2 Hz to 17.0±2.7 Hz) and particle transport speed (38.9±4.6 µm/s to 83.4±8.3 µm/s) to an extent that was comparable to a supramaximal dose of ATP. The increase in particle transport speed was totally prevented by methysergide (100 µM). Blockade of muscarinic receptors by atropine (1 µM) did not reduce the effect of serotonin, although it was effective in preventing the increase in particle transport speed mediated by muscarine (100 µM). Immunohistochemistry demonstrated serotonin in mast cells pointing towards mast cells and platelets as possible endogenous sources of serotonin.

**Conclusions/Significance:**

These results indicate that serotonin is a likely endogenous mediator that can increase cilia-driven transport independent from acetylcholine during activation of mast cells and platelets.

## Introduction

The transport of mucus and inhaled particles such as dust and bacteria is pivotal to clean the airways and prevents the accumulation of noxious substances and particles which could otherwise lead to disease [1]. This directed transport is maintained by ciliated cells in the airway epithelium. They drive mucus and particles by coordinated beating of the cilia towards the orifice of the larynx to be either expectorated or swallowed [2]. Several neuronal mediators such as acetylcholine, noradrenalin, and substance P are known to influence ciliary beat frequency and, thus, cilia-driven transport [2].

Serotonin is a biogenic amine that functions as a neurotransmitter in invertebrates and vertebrates and can be released from non-neuronal cells in the lung [3], [4]. Although the involvement of serotonin in regulating ciliary beat frequency in the airways of vertebrates is not clear, it clearly regulates various aspects of lung function under physiological as well as pathophysiological situations. In the pulmonary vasculature, serotonin induces vasoconstriction and is implied in pulmonary hypertension [5]. It also leads to contraction of airway smooth muscle if released from mast cells in situ or applied exogenously in vitro [6]–[8], and is implied in asthma pathogenesis in a mouse model of asthma [9]. Serotonin also contributes to an increase of periciliary liquid and, thus, potentially protects the airway epithelium by directing water towards the airway lumen by facilitating mucociliary clearance [10]. It has been suggested that serotonin induces the release of acetylcholine from mouse airway epithelial cells [11]. Since acetylcholine is known to increase clilary beat frequency, the release of acetylcholine might be a serotonin-driven mechanism to increase ciliary beat frequency in vertebrates.

In invertebrates, the ciliostimulatory role of serotonin is firmly established [12]–[14]. However, to date, the role of serotonin in the regulation of ciliary beat frequency and cilia-driven particle transport in airways of vertebrates is not clear. Reports describe serotonin as ciliostimulatory or cilioinhibitory in the rabbit trachea [15], [16] or without effect in the frog palate preparation and in the rat trachea [14], [17].

Here, we addressed this issue by investigating the effect of serotonin on ciliary beat frequency and particle transport speed using the acutely explanted mouse trachea as a model. Use of appropriate blockade of cholinergic pathways served to examine a possible involvement of acetylcholine in the effects evoked by serotonin. Endogenous sources of serotonin in the mouse trachea were determined using immunohistochemistry.

## Materials and Methods

### Animals

C57/Bl6 Mice of both sexes were used in this study. The experiments were done according to the German guidelines for the care and use of laboratory animals.

### Preparation of the trachea and imaging

Mice were killed by inhalation of isoflurane (Baxter, Unterschleißheim, Germany). The thorax was opened and the submandibular gland and the infrahyoid musculature were removed. The trachea was cut caudal to the larynx and cranial to the bifurcation. Then, the trachea was removed and transferred to a Delta T culture dish (Bioptechs, Butler, PA, USA) whose bottom was covered with a thin film of Sylgard polymer (Dow Corning, Wiesbaden, Germany) and that was filled with 2 ml cooled Hepes-Ringer solution. Connective tissue and blood vessels were removed, and the trachea was oriented with the m. trachealis facing upward and fixed with two insect needles.

The m. trachealis was cut using Vannas-Tübingen spring scissors (FST, Heidelberg, Germany). Then, the Hepes-Ringer solution was replaced by 1.5–2 ml fresh buffer and the culture dish was transferred to the Delta T Stage holder 30 min after the animal's death. The dish was heated to 30°C and this temperature was held constant during the experiment. Imaging was done with a Till Vision imaging system (Till Photonics, Gräfelfing, Germany) based on an Olympus BX50 WI microscope (Olympus, Hamburg, Germany).

**Figure 1:**
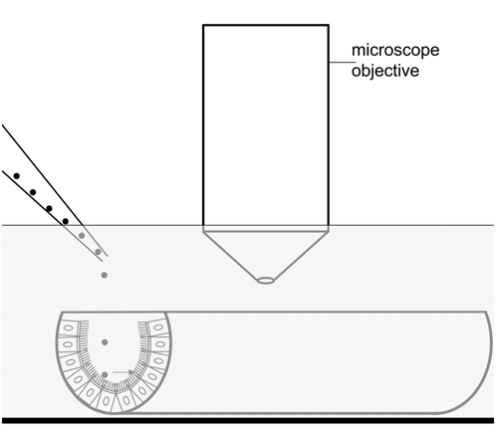
Schematic drawing of the setup used to measure cilia-driven particle transport speed and ciliary beat frequency. The trachea is submerged and the epithelial surface is observed using a dip-in objective.

Prior to measurements, 4 µl of a polysterene bead suspension (mean diameter 2.8 µm or 4.5 µm; Dynal Biotech GmbH, Hamburg, Germany) were added. Then, the epithelial surface of the trachea was imaged in bright field mode using a 20× water immersion objective (Olympus, Figure 1). Transported Dynabeads were readily identified by their appearance and their brownish color. An area between two cartilages was chosen for recordings to prevent large differences in image brightness. For each time point 200 images were taken with an exposure time of 20 ms and a delay of 85 ms between two images. To validate the proper function of the cilated cells in the airway epithelium, ATP, which stimulates ciliary beat frequency via purinergic receptors, was applied at the end in most experiments. A total of 41 mice were used for the particle tracking experiments (5 to 6 tracheae from 5 to 6 mice for each experiment). The exact number of mice used for each experiment is given in the figure legends.

To measure ciliary beat frequency, the epithelial surface was visualized using a 40× dip in objective (Zeiss, Jena, Germany) and an EHD SMX-150M CMOS camera (EHD Imaging, Damme, Germany). One thousand images (100 images/s) were taken per time point. For measuring ciliary beat frequency, 40 cells from 4 mice (10 cells/animal) were examined.

### Drugs

The following substances were used: ATP, muscarine, serotonin, methysergide, all from Sigma, Deisenhofen, Germany, and atropine, from Braun, Melsungen, Germany. Drugs were pipetted in the organ bath and the buffer was mixed gently with a pipette.

### Particle Tracking

To track individual particles, the following image processing was performed: To remove non-moving objects in the images, an average image of the 200 images of an individual series was calculated and subtracted on a pixel-by-pixel basis from each image in the series. If the value of a subtraction got negative, the absolute value was taken. Through this procedure, the image of the formerly darker polysterene beads became bright. Then, a copy of this series was changed to a binary picture by a thresholding procedure so that the image of polysterene beads was set to bright and the background was set to dark. The original film was reduced from 12 bit to 8 bit greyscale and both series were used to track the particles by an automatic tracking procedure using the TILLvisTRAC software (Till Photonics). Only tracks that were measured over a length of at least 10 frames were included in further calculations.

Of all tracks that fulfilled this criterion, the average of the mean speed was calculated.

### Determining ciliary beat frequency

The ciliary beat frequency for each time point was determined by analyzing the change in mean brightness over time in a small area of an individual ciliated cell by fast Fourier transformation using Autosignal 1.7 (SeaSolve Software Inc., San Jose, CA, USA).

### Statistical analysis

Comparison within one time series was performed using a global Friedman test followed by a Wilcoxon test to compare selected time points. To compare the same time point from different experiments, analysis was carried out by using the Kruskal-Wallis test for global comparison followed by the Mann-Whitney test to compare individual experiments. P values<0.05 were regarded as being statistically significant.

### Scanning electron microscopy

After particle tracking experiments (n = 5), tracheae were pinned on small cork plates to prevent shrinkage, fixed with Monti's fixative (2% glutardialdehyde, 0.6% paraformaldehyde, and 0.03% CaCl_2_ in 0.06 M cacodylate buffer, pH 7.35 [18]), washed in cacodylate buffer, dehydrated in increasing concentrations of acetone, critical point dried, sputtered with gold and evaluated using a scanning electron microscope.

### Staining of mast cells with toluidine blue

Tracheae were fixed in 4% PFA in PBS, washed in distilled water for 2×10 min, permeabilized with acetone for 10 min, washed for 3×5 min in distilled water, incubated in 0.1 M HCl for 5 min and stained for 15 min in toluidine blue in 1 M HCl. Then, the tissue was washed in 0.1 M HCl for 2×5 min and kept for several hours in 0.1 M HCl until the staining of the cartilage was removed.

### Preparation of semithin sections

Mice (n = 3) were killed by inhalation of an overdose of isoflurane and fixed by transcardiac perfusion with Monti's fixative, preceded by rinsing with 0.9% NaCl in distilled water. Tracheae were removed, dehydrated in increasing alcohol concentrations and embedded in araldite. Semithin sections were cut and stained with Richardson's stain.

### Immunohistochemistry

Antibodies and their sources were as follows: anti-serotonin (1∶50, mouse monoclonal, clone 5HT-H209, Dako, Hamburg, Germany) which was labelled prior to incubation using an Alexa 555 Zenon kit according to the instructions of the manufacturer (Invitrogen, Karlsruhe, Germany).

Mice were killed by inhalation of an overdose of isoflurane and fixed by transcardiac perfusion of 4% paraformaldehyde in 0.1 M phosphate buffer, pH 7.2, preceded by rinsing solution [19]. Tracheae were cryoprotected using 18% sucrose and snap frozen. Cryosections (10 µm) were cut, air-dried and incubated for 1 h in 10% normal horse serum containing 0.5% Tween 20, 0.1% BSA in PBS, pH 7.4.. The labeled anti-serotonin antibody was subsequently diluted in 0.005 M phosphate buffer containing 0.01% NaN_3_ and 4.48 g/l NaCl, and applied for 1 h at 37°C. Then, sections were fixed with 4% paraformaldehyde in 0.1 M phosphate buffer, washed again and coverslipped in carbonate-buffered glycerol (pH 8.6).

Sections were evaluated with an epifluorescence microscope using appropriate filter sets.

## Results

### Measuring Cilia-driven particle transport to assess the coordinated function of ciliated cells

**Figure 2:**
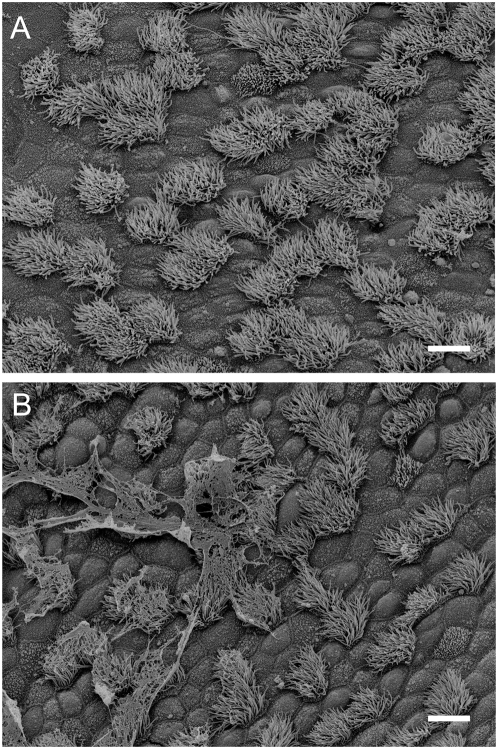
Scanning electron microscopy of the tracheal epithelium after the experiment. A. No continuous mucus layer can be detected on the epithelial surface after the experiments. B. Occasionally, small amounts of mucus are found on the epithelium . Small mucus-particle aggregates were also occasionally observed during measurements of particle transport speed (compare Movie S2). Bars = 10 µm.

In the submerged trachea, particles were transported cranially by coordinated beating of ciliated cells. Transport was not dependent on the size of the particles (see Movie S1) and did not involve a continuous mucus layer as verified by examining tracheae after the experiment using scanning electron microscopy (Figure 2A). Occasionally, small amounts of mucus were present and entrapped particles which were dragged along the epithelial surface (see Movie S2). Small areas that were covered with mucus were occasionally detected by scanning electron microscopy after the experiment (Figure 2B).

**Figure 3:**
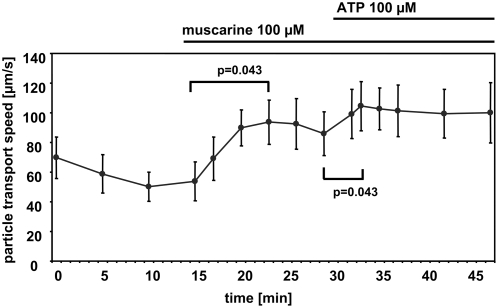
Muscarine and ATP increase cilia-driven particle transport speed. Muscarine increases cilia-driven particle transport speed which can be further increased by ATP. Statistical analysis: Global Friedman test followed by a Wilcoxon test to compare selected time points. Mean±S.E.M. is shown. N = 5 tracheae from 5 animals. P values below 0.05 are set in bold type.

The established stimuli of ciliary beat frequency, muscarine and ATP, increased cilia-driven particle transport (Figure 3).

### Serotonin increases cilia-driven particle transport

**Figure 4:**
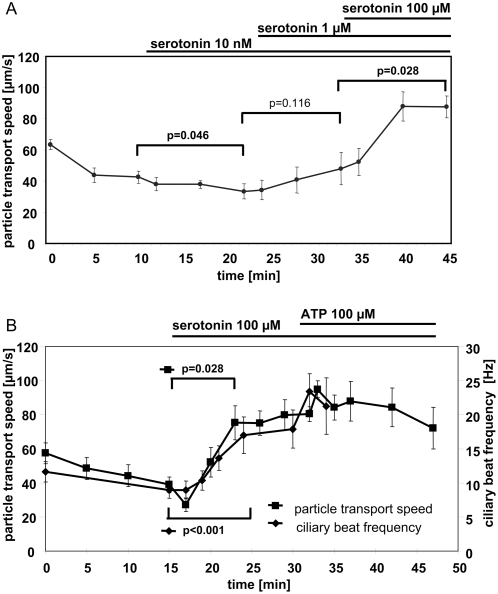
Serotonin increases particle transport speed and ciliary beat frequency. A. Cumulative increase of serotonin concentration increases cilia-driven particle transport speed. Statistical analysis: global Friedman test followed by a Wilcoxon test to compare selected time points. Mean±S.E.M. is shown. N = 6 tracheae from 6 animals. P values below 0.05 are set in bold type. B. The increase in cilia-driven particle transport speed is paralleled by an increase in ciliary beat frequency. Mean±S.E.M. is shown. Statistical analysis: Global Friedman test followed by a Wilcoxon test to compare selected time points. N = 6 tracheae from 6 animals for the measurement for particle transport speed, and n = 40 cells from 4 tracheae from 4 animals. S.E.M. was calculated using the number of animals. P values below 0.05 are set in bold type.

Cumulative application of 10 nM and 1 µM serotonin did not increase the particle transport speed significantly over the initial speed prior to stimulation. Stimulation with 100 µM serotonin increased the particle transport speed from 47.9±10.4 µm/s (33 min) to 87.5±7.0 µm/s (45 min; p = 0.028; Figure 4A). The concentration of 100 µM serotonin was used in further experiments.

### Serotonin increases cilia-driven particle transport by increasing cilary beat frequency

Serotonin (100 µM) increased particle transport speed from 38.9±4.6 µm/s (15 min) to 83.4±8.3 µm/s (23 min; p = 0.028, Figure 4B). Stimulation with a supramaximal dose of ATP (100 µM) increased the particle transport speed further to 94.8±4.9 µm/s (23 min). Serotonin (100 µM) also increased ciliary beat frequency from 8.9±1.2 Hz (15 min) to 17.0±2.7 Hz (25 min) and to 23.3±2.7 Hz 2 min after addition of ATP (Figure 4B).

### Methysergide blocks the serotonin-induced increase in cilia-driven particle transport

**Figure 5:**
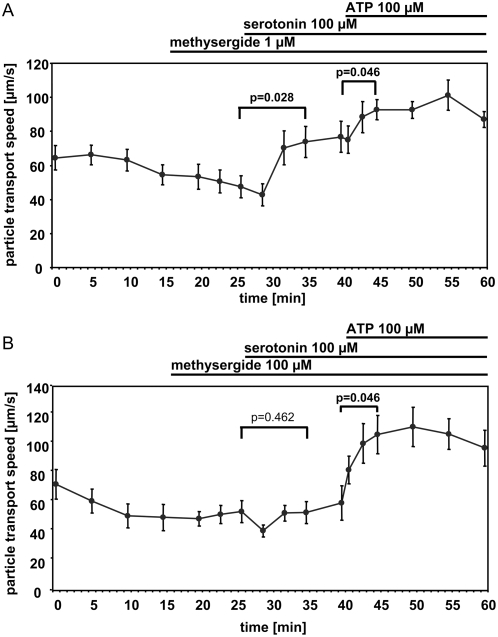
The serotonin-induced particle transport speed can be completely blocked by 100 µM methysergide. A. Methysergide (1 µM). B. Methysergide (100 µM). Statistical analysis: Global Friedman test followed by a Wilcoxon test to compare selected time points. Mean±S.E.M. is shown. N = 6 tracheae from 6 animals for each experiment. P values below 0.05 are set in bold type.

Prior incubation with 1 µM methysergide did neither reduce the serotonin-induced increase in particle transport speed nor influence the ATP-mediated increase (Figure 5A). Incubation with 100 µM methysergide fully prevented the serotonin-induced increase in particle transport speed but did not influence the ATP-mediated increase in particle transport speed (Figure 5B).

### Atropine blocks the muscarine- but not the serotonin-induced increase in cilia-driven particle transport

**Figure 6:**
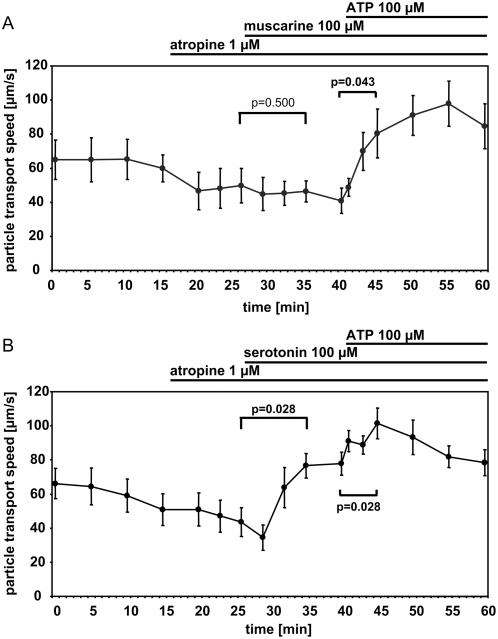
Atropine blocks the ciliostimulatory effect of muscarine but not of serotonin. A. Muscarince (100 µM). B. Serotonin (100 µM). Statistical analysis: Global Friedman test followed by a Wilcoxon test to compare selected time points. Mean±S.E.M. is shown. N = 6 tracheae from 6 animals for each experiment. P values below 0.05 are set in bold type.

The increase in particle transport speed by 100 µM muscarine was totally prevented using 1 µM atropine without attenuating the response to ATP (Figure 6A). In contrast, the same concentration of atropine failed to block the serotonin-induced increase in particle transport speed (Figure 6B), indicating that serotonin does not increase particle transport speed via release of acetylcholine.

### Serotonin-immunoreactivity is present in mast cells

**Figure 7:**
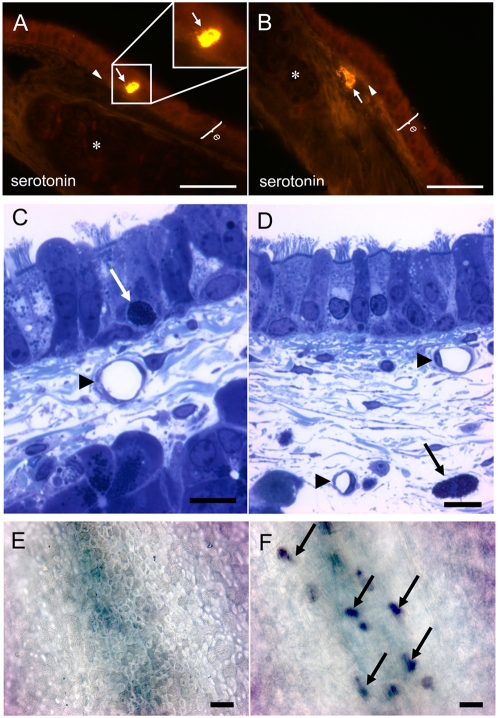
Mast cells contain serotonin in the mouse trachea. A, B. Fluorescence microscopy. Serotonin-immunolabeling is located in mast cells (A and B; arrow) Epithelium = e; cartilage = *. C, D. The presence of mast cells in the epithelium and in the lamina propria is shown in semithin sections. Arrows = mast cells, Arrowheads = capillaries. E, F. Toluidine blue staining of subepithelial mast cells in a trachea whole-mount. E. View from the lumen onto the epithelium. F. Same area as in E but with the focal plane below the epithelium. Arrows = toluidine blue-stained mast cells. Bars in A, B = 50 µm, in C, D = 10 µm, in E, F = 20 µm.

Serotonin-immunoreactivity was observed in mast cells in the lamina propria of the mouse trachea (Figure 7A, B), identified by numerous granula (Figure 7A inset). The labelling intensity was higher in mast cells found in the epithelial layer than in mast cells that were located under the basal membrane in the connective tissue in close proximity to blood vessels. In vessels that still contained blood, serotonin-immunoreactivity was found in platelets (not shown).The presence of mast cells in the subepithelial layer and in the epithelium was validated in conventional semithin sections (Figure 7C, D) and in whole mount preparations of tracheae using toluidine blue (Figure 7E, F).

## Discussion

In this study, we have shown that serotonin increases cilia-driven particle transport in the mouse trachea.

Often, ciliary beat frequency is used as a surrogate measurement for cilia-driven transport. In this study, we primarily focused upon particle transport speed because it not only informs about the function of individual ciliated cells but takes into account the coordinated function of many cells and thus more closely reflects the in vivo situation. Since our preparation utilized uniformly sized round particles and was devoid of a mucus blanket, the measurements primarily reflect the function of the ciliated cells and not the composition of mucus and/or the form of the particles.

Serotonin is known to increase ciliary beating in a variety of species such as in gills of mussels [20], in sea urchin larvae [12], and in embryos of the mollusk helisoma trivolvis [13], [21]. The concentration of serotonin that induced a large effect in particle transport speed in our setup is the same that was maximally effective in rat ependymal cells [22] as well as in helisoma trivolvis [13], indicating that serotonin is a well conserved regulator of ciliated cell activity.

The observed increase in particle transport speed in the mouse trachea was due to an increase in ciliary beat frequency. An earlier study indicated that serotonin reduces ciliary beat frequency [15]. Differences in experimental setup may account for this discrepant observation. In that study, serotonin was given intravenously in an anaesthetized animal and caused strong vasoconstriction in the trachea as judged by the description of “abrupt blanching of the tracheal mucosa” and a “characteristic bloodlessness” after intracardial serotonin application. Thus, the decreased ciliary beat frequency likely reflects a general impairment of epithelial function due to impaired blood supply which is underpinned by the observation of enhanced vulnerability to trauma of the epithelium in this study [15].

In our experiments, methysergide selectively blocked the effect of serotonin on ciliary beat frequency. Of note, intranasal methysergide was also effective in reducing airway inflammation and remodeling in a mouse chronic asthma mouse model and it largely reduced the allergen mediated early response in sensitized rats [9], [23] underpinning that serotonin is released in vivo during allergen challenge in substantial amounts. Since methysergide is known to block a rather breoad spectrum of serotonin receptors (at least 5-HT1, 5-HT2, and 5HT-7 receptors) it can not be deduced which receptor is mediating the increase in ciliary beat frequency and particle transport speed. It is possible that more than one receptor subtype is involved as it is the case for muscarinic receptors [24].

The increases in cilary beat frequency and particle transport speed in response to serotonin had a delay of 4–5 minutes, indicating that serotonin might not act directly on ciliated cells but via release of mediators from other cells. In a recent paper, serotonin was assumed to evoke release of acetylcholine from the airway epithelium which in turn shall mediate bronchoconstriction [11]. Since acetylcholine also increases ciliary beat frequency and particle transport speed via activation of muscarinic receptors [24], [25], we also explored the possibility that the increase in cilia-driven particle transport was mediated by release of acetylcholine from the airway epithelium and subsequent paracrine activation of muscarinic receptors. However, blockade of muscarinic receptors with atropine in a concentration sufficient to fully block the effect of 100 µM muscarine had no effect on serotonin-induced increase of particle transport speed in our model. Therefore, a significant participation of acetylcholine in the serotonin-induced increase in particle transport speed can be ruled out. However, it is possible that other yet to be determined substances mediate the serotonin effect on ciliated cells.

Serotonin is found in murine mast cells and platelets [26], [27] which are both present in the mouse trachea. Other possible sources of serotonin could be neuroendocrine cells and/or nerve fibers. However, we failed to locate cells or structures resembling neuroendocrine cells or nerve fibers that were serotonin-immunoreactive making them an unlikely source for serotonin.

Mast cells can be activated by cross linking of IgEs bound to the high affinity IgE receptor on their surface by antigen such as allergens [28]. This cross linking leads to the release of mast cell granules that contain serotonin. Serotonin released upon antigen stimulation can lead to constriction of the tracheal muscle [8]. It is known that substances released from the airway epithelium can influence airway smooth muscle even in larger animals such as guinea pigs [29]. Thus, it is likely that serotonin released from mast cells can also reach the airway epithelium. Since mast cells of rodents harbor much higher concentrations of serotonin than human mast cells [30], this mechanism is likely to be present predominantly in rodents.

Another ubiquitous source of serotonin that is shared by rodents and humans are platelets. They release serotonin after activation. Normally, thrombocyte activation is associated with blood vessel damage. However, recent data indicate that platelets are also activated during asthma. Interestingly, platelets also contain the low and the high affinity IgE receptor and can be activated in allergic patients through the specific allergen [31]. Since arterioles, capillaries, and venules are found immediately underneath the airway epithelium, serotonin released from platelets could also reach the airway epithelium especially when postcapillary venules and capillaries become leaky during inflammation and thereby increase cilia-driven particle transport. This view is supported by the fact that that intravenously given Evans Blue can be readily detected in the bronchioalveolar lavage fluid after allergen challenge in rats [32]. Of note, Fernandez-Gonzalez and coworkers have shown in a recent study that disruption of ciliary function in mice drastically augments the pathology after allergen challenge [33]. This indicates that allergen removal is an important mechanism to limit airway inflammation. Acutely, the physiologic function of serotonin released from mast cells or platelets could be to locally increase ciliary beat frequency. Together with the described action of serotonin to increase periciliary liquid volume [10], release of serotonin may effectively accelerate the removal of unwanted substances/particles from the airway epithelium.

In conclusion, our data show that serotonin increases ciliary beat frequency as well as cilia-driven particle transport independently from acetylcholine by a mechanism sensitive to the serotonin receptor antagonist, methysergide. Since serotonin can be released in rodents and man from several cell types the airways, it is likely that serotonin is an endogenous activator of mucociliary transport accelerating removal of unwanted substances from the surface of the airway epithelium especially in the context of acute inflammation.

## Supporting Information

Movie S1 The movie is showing a large polysterene particle (ca. 20 µm diameter) that is transported together with smaller particles (4.5 µm mean diameter) by the ciliated epithelium.
(0.62 MB MOV)

Movie S2 The movie is showing particles that are trapped in mucus and are transported together on the left hand side. Particles on the right hand side are being transported individually.
(3.08 MB MOV)
